# Long-term efficacy and safety of sirolimus for retinal astrocytic hamartoma associated with tuberous sclerosis complex

**DOI:** 10.3389/fcell.2022.973845

**Published:** 2022-11-18

**Authors:** Chen-Xi Zhang, Kai-Feng Xu, Qin Long, Xiao Zhang, Zhi-Kun Yang, Rong-Ping Dai, Zhi-Qiao Zhang

**Affiliations:** ^1^ Department of Ophthalmology, Key Laboratory of Ocular Fundus Diseases, Peking Union Medical College Hospital, Chinese Academy of Medical Sciences and Peking Union Medical College, Beijing, China; ^2^ Department of Pulmonary and Critical Care Medicine, State Key Laboratory of Complex Severe and Rare Diseases, Peking Union Medical College Hospital, Chinese Academy of Medical Sciences and Peking Union Medical College, Beijing, China

**Keywords:** long-term, retinal astrocytic hamartoma, sirolimus, tuberous sclerosis complex, treatment

## Abstract

Mammalian target of rapamycin (mTOR) inhibitors (sirolimus or everolimus) have been demonstrated effective in reducing the size of tuberous sclerosis complex (TSC)-associated retinal astrocytic hamartoma (RAH) in short term. To investigate the long-term efficacy and safety of sirolimus on TSC-associated RAH, 13 TSC-associated RAH patients (59 RAH lesions) who received sirolimus therapy for at least 2 years were retrospectively enrolled in this study. Changes in the maximal thickness (MT) of RAH on optical coherence tomography and the longest base diameter (LBD) of RAH on color fundus photography were assessed. The results showed that for a mean follow-up of 39 months, sirolimus was associated with a mean reduction of 14.6% in MT and 6.8% in LBD of RAHs. The main impacts of sirolimus occurred within the first 6–12 months, with 14.8% reduction in MT and 4.7% reduction in LBD. Mouth ulceration (10 [76.9%]) and acne (9 [69.2%]) were the most common adverse events. These follow-up data support the long-term use of sirolimus in TSC-associated RAH patients, and persistent use of sirolimus possibly prevents tumor regrowth.

## Introduction

Tuberous sclerosis complex (TSC) is a genetic disorder characterized by multi-system hamartomas, which occur from mutations of the TSC1/TSC2 genes and subsequent overactivation of the mammalian target of rapamycin (mTOR) signaling pathway and dysregulation of cell proliferation (Orlova and Crino, 2010). mTOR inhibitors, such as sirolimus and everolimus, have been demonstrated effective in the treatment for several TSC-related lesions, including subependymal giant cell astrocytomas (SEGAs), renal angiomyolipomas (AMLs), and pulmonary lymphangioleiomyomatosis (LAM) (Curatolo et al., 2016).

Retinal astrocytic hamartoma (RAH), as the most common ophthalmic manifestation in TSC, occurs in 50–80% of individuals with TSC (Hunt, 1983; Kiribuchi et al., 1986; Robertson, 1999; Rowley et al., 2001). Most RAHs remain stable over years, but aggressive growth leading to severe ocular complications, including retinal detachment and neo-vascular glaucoma, was described in a small RAH series (Shields et al., 2004). In our previous report, sirolimus was shown to reduce the size of RAHs in an average 8-month follow-up (Zhang et al., 2015). Similar short-term effects were also observed in the other mTOR inhibitor (everolimus) for TSC-associated RAHs (Nallasamy et al., 2017; Marciano et al., 2018).

However, the long-term effects of mTOR inhibitors for RAHs are unclear. Whether mTOR inhibitor therapy could result in sustainable reduction or stabilization of the RAH size over a longer follow-up period is still unknown. Considering the therapy of mTOR inhibitors is always in need of long-term use in TSC patients, it is necessary to investigate the long-term efficacy and safety of mTOR inhibitors on TSC-associated RAH (Tran and Zupanc, 2015). Therefore, we performed a retrospective study to assess the efficacy and safety of sirolimus for TSC-associated RAH over at least 2 years.

## Materials and methods

To determine the long-term efficacy and safety of sirolimus for RAHs, we retrospectively reviewed the medical records of all the patients with a definite diagnosis of TSC who were referred to the Department of Ophthalmology, Peking Union Medical College Hospital for the evaluation of RAH from 1 August 2012 to 31 January 2021. Eligible patients met the following inclusion criteria: (1) patients aged 10–60; (2) presence of TSC-associated RAHs; (3) treatment with sirolimus for subependymal giant cell astrocytomas, renal angiomyolipomas, or pulmonary lymphangioleiomyomatosis; (4) sirolimus therapy with ophthalmic baseline evaluation (performed within 1 month prior or after the initiation of sirolimus therapy); (5) sirolimus therapy with an ophthalmic follow-up examination available over at least 24 months; and (6) at least one RAH documented by spectral-domain optical coherence tomography (SD-OCT) and/or fundus photography with follow-up data. Exclusion criteria included (1) history of any eye treatment for RAHs and (2) history of mTOR inhibitor treatment. The protocol of this study was approved by the ethical committee of Peking Union Medical College Hospital (Approved number: S-K009), and the study was conducted in compliance with the Declaration of Helsinki. Sirolimus (Rapamune, Pfizer, Rye Brook, NY) was administered orally starting at 1 or 2 mg daily and adjusted subject to tolerability to achieve a targeting serum concentration of 5–10 ng/ml; adverse events were monitored for safety assessment.

In total, 13 eligible patients were enrolled. These patients were followed mostly at 6–12 months interval, and the last available assessment was conducted after 24 months of sirolimus therapy. At each visit, a complete ophthalmic examination was performed, including best-corrected visual acuity, slit-lamp examination, color fundus photography by TRC NW6S (Topcon Corp, Japan), and optical coherence tomography (OCT). The OCT device used for almost all the visits was SD-OCT (3D OCT 1000 Mark II, Topcon, Inc, Tokyo, Japan), except of the last visit in two patients which was assessed by swept-source optical coherence tomography (SS-OCT, DRI-OCT Triton, Topcon, Inc, Tokyo, Japan). We collected the baseline and follow-up data comprising maximal thickness (MT) of RAH on OCT and the longest base diameter (LBD) of RAH on color fundus photography. The MT, as the primary efficacy outcome, was defined as the distance from the highest peak of the anterior surface of RAH to the retinal pigment epithelium after reviewing all OCT B-scans. The LBD of RAH on fundus photography was measured and expressed in optic disc diameter (DD) units, reflecting the change of the RAH size in horizontal length. Limited by RAH location and eye rotation, only those fully scanned by OCT or clearly identified by fundus images at given follow-up periods were involved in the corresponding MT or LBD analysis. RAHs were classified into three morphological groups, with type 1 showing semitransparent, non-calcified, flat appearance, type 2 characterized by mulberry-like multinodular calcification, and type 3 combining features of both types 1 and 2 (Robertson, 1999).

Statistical evaluation was performed to compare the baseline data with post-treatment data using the paired t-test. Factors related to sirolimus efficacy on the RAH size were investigated by one-way ANOVA. Differences between RAHs eligible for MT or LBD analysis and those ineligible were studied using one-way ANOVA or the Fisher exact test. *p* < 0.05 was considered statistically significant. Data analyses were performed in SPSS V.23 (IBM, New York, United States).

## Results

In total, 13 TSC patients (6 men and 7 women) with a mean age of 24.6 ± 9.1 years (range, 13–42; median, 24) at baseline were enrolled. The mean follow-up was 39.0 ± 8.7 months (range, 28–55; median, 36). A total of 59 RAHs were detected in 24 eyes of the 13 patients, which were bilateral in 84.6% (11/13) of the patients (Table 1). Of the 24 affected eyes, 13 had two or more RAHs. The mean best-corrected visual acuities at baseline and the last visit were 0.05 ± 0.16 and 0.04 ± 0.18 logarithm of the minimum angle of resolution (logMAR) respectively, with no significant difference being revealed (*p* = 0.68, paired t-test, equivalent to snellen visual acuity 20/22 ± 20/29, and 20/22 ± 20/30, respectively). Of the 59 RAHs, 54 (91.5%) were type 1 lesions and 5 (8.5%) were type 3. No type 2 RAH was found in these enrolled patients or any type transition was observed in the follow-up. These RAH lesions were most commonly seen in the superotemporal quadrant (35.6%, 21/59), followed by inferotemporal (16.9%, 10/59), superonasal (15.3%, 9/59), inferonasal (15.3%, 9/59), perifoveal (13.6%, 8/59), and peripapillary (3.4%, 2/59) quadrants.

**TABLE 1:** Patient baseline characteristics.

	Patient (N = 13)
Median age (range), years	24 (13–42)
Median follow-up (range), months	36 (28–55)
Male, n (%)	6 (46.2)
TSC-involved organs, n (%)	
Eye	13 (100)
Skin	13 (100)
Brain	12 (92.3)
Kidney	12 (92.3)
Lung	8 (61.5)
Bilateral RAH, n (%)	11 (84.6)
No. of RAH lesions, n (%)	
1–2	6 (46.2)
3–5	4 (30.8)
>5	3 (23.0)
Median MT of RAH (range), μm	541 (369–878)^a^
Median LBD of RAH (range), DD	0.74 (0.13–1.75)^a^

^a^ Calculated in RAHs eligible for MT or LBD analysis.

TSC, tuberous sclerosis complex; RAH, retinal astrocytic hamartoma; MT, maximal thickness; LBD, longest base diameter; DD, disc diameter.

### Maximal thickness of retinal astrocytic hamartoma on optical coherence tomography

**FIGURE 1:**
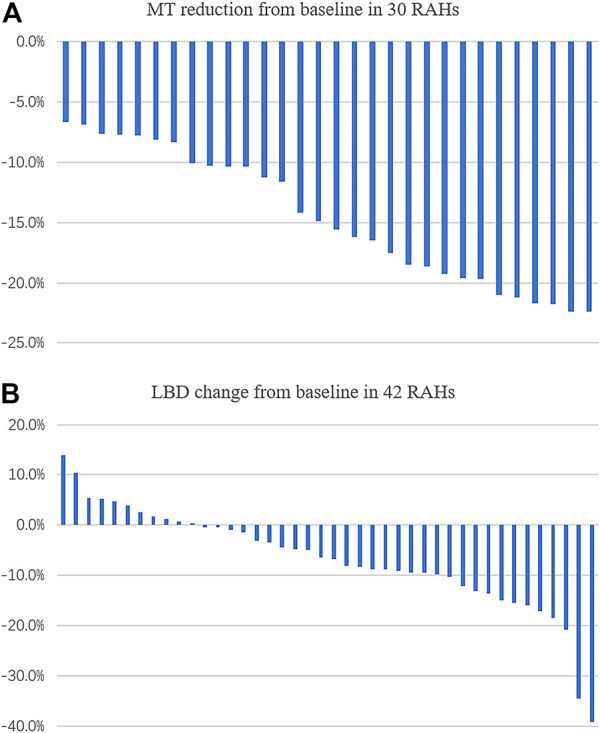
Change (%) in maximal thickness (MT), the longest base diameter (LBD) of retinal astrocytic hamartoma (RAH) after sirolimus therapy for over 2 years. **(A)** MT was found reduced in all the eligible lesions. **(B)** LBD reduction was observed in 73.8% (31/42) of RAH lesions.

Of the 59 RAHs, only 30 RAH lesions were fully scanned by OCT both at baseline and the last visit. The mean MT of RAHs at the first and last visit were 547.87 ± 113.76μm and 465.27 ± 87.63 μm, respectively (*p* < 0.001, paired t-test), and the mean MT reduction was 14.6%, with MT being reduced by ≥ 10% from baseline in 76.7% (23/30) RAHs, ≥ 20% reductions in 20.0% (6/30). No RAHs presented an increase in MT from baseline on SD-OCT (Figure 1A). No significant correlation was observed between lesion reduction and the RAH type (*p* = 0.78, one-way ANOVA) or RAH location (*p* = 0.085, one-way ANOVA) (Supplementary Table S1). Comparing the 30 RAH lesions with the other 29 RAH lesions ineligible for MT analysis, no significant differences were found in age (*p* = 0.24, one-way ANOVA), follow-up period (*p* = 0.30, one-way ANOVA), RAH type (*p* = 0.35, Fisher exact test), and RAH location (*p* = 0.48, Fisher exact test) (Supplementary Table S2).

### Longest base diameter of retinal astrocytic hamartoma on color fundus photography

We managed to measure the LBD at baseline and at the last visit in 42 of the 59 RAHs. The mean LBD of RAHs at the first and last visit were 0.82 ± 0.38DD and 0.76 ± 0.37DD, respectively (*p* < 0.001, paired t-test). The mean change of RAH LBD was –6.8% (Figure 1B). No significant correlation was observed between the LBD change and RAH type (*p* = 0.85, one-way ANOVA) or RAH location (*p* = 0.18, one-way ANOVA) (Supplementary Table S3). A total of 17 RAH lesions ineligible for LBD analysis did not significantly differ from the 42 RAH lesions in age (*p* = 0.70, one-way ANOVA), follow-up period (*p* = 0.62, one-way ANOVA), RAH type (*p* = 0.55, Fisher exact test), and RAH location (*p* = 0.24, Fisher exact test) (Supplementary Table S4).

### Short-term (6–12 m) vs. long-term effects of sirolimus for retinal astrocytic hamartoma

**FIGURE 2:**
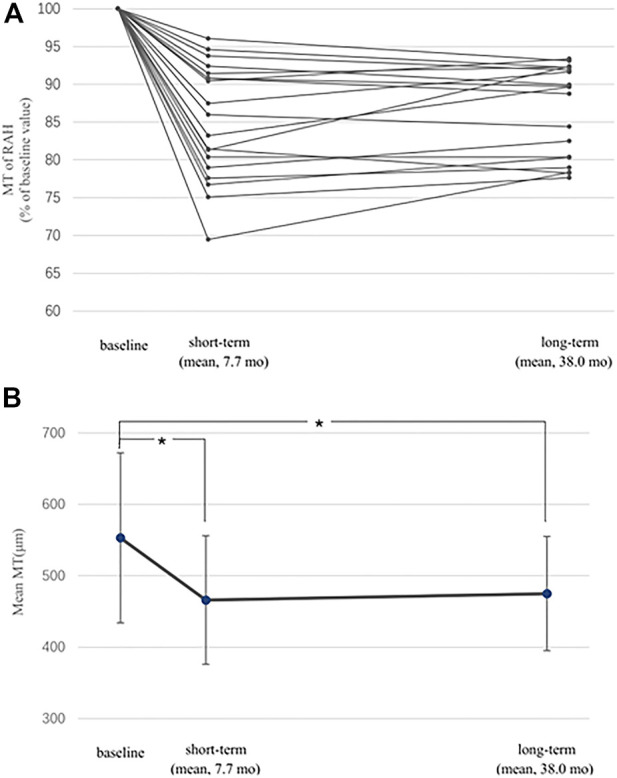
Short-term and long-term effects of sirolimus on maximal thickness (MT) of retinal astrocytic hamartoma (RAH). **(A)** MT of 19 eligible RAHs at follow-ups is expressed as a percentage of the baseline MT. MT reductions were observed in all lesions for both short-term and long-term follow-ups. **(B)** Significant difference was found when comparing mean MT of RAHs at the short-term/long-term follow-up with the baseline value (*, *p* < 0.001). I bars indicate the standard errors.

We compared the short-term and the long-term efficacy of sirolimus for RAH associated with TSC based on data from baseline, 6–12 months after sirolimus treatment (short-term), and the last visit (≥ 24 m, long-term). MT at short-term was available in 19 of the 30 RAH lesions, of which the mean short-term follow-up was 7.7 ± 2.0 months, while that of the long-term follow-up was 38.0 ± 8.2 months. A significant MT decrease was revealed by the paired t-test in the short-term follow-up (mean baseline MT 553.1 ± 119.9 μm, mean short-term MT 466.4 ± 90.0 μm, and *p* < 0.001), and this benefit persisted in the long-term follow-up (mean long-term MT 475 ± 88.9 μm, *p* < 0.001) (Figure 2). No significant difference was observed in MT between the short-term and long-term follow-up (*p* = 0.12, paired t-test). As to the RAH LBD on fundus photography, 28 of the 42 RAH lesions with 6–12 m follow-up were measured (mean short-term follow-up 7.6 ± 2.1 months; mean long-term follow-up 37.1 ± 7.2 months). Similarly, a significant reduction in RAH LBD was initially observed in the short-term follow-up (mean baseline LBD 0.81 ± 0.38 DD, mean short-term LBD 0.77 ± 0.35 DD, and paired t-test, *p* = 0.01) and lasted to the final visit (mean long-term LBD 0.77 ± 0.37 DD, paired t-test, and *p* = 0.002). There is no significant difference between short-term and long-term efficacy of sirolimus on RAH LBD (*p* = 0.61, paired t-test).

### Adverse effects

For the 13 enrolled TSC patients, the average blood sirolimus concentration was 6.0 ± 2.9 ng/ml (range, 3.0–14.0 ng/ml). Sirolimus was well-tolerated for the long-term follow-up. Mouth ulceration (10 [76.9%]) and acne (9 [69.2%]) were the two most frequently reported adverse events in this study, followed by menstrual abnormality (4 [30.8%]) and diarrhea (1 [7.7%]). No severe adverse effects were observed during follow-up periods; neither were ocular complications. One patient required temporal interruption of sirolimus treatment due to diarrhea and restarted the treatment after 1 month. No patients terminated the use of sirolimus because of adverse effects.

## Discussion

Sirolimus, as an mTOR inhibitor, was isolated in 1973 from soil samples in Easter Island. It was first used as an immunosuppressive agent for prophylaxis of organ rejection. And for decades, it has demonstrated its effectiveness in treating TSC-related manifestations. With sirolimus treatment, tumor shrinkage was observed in SEGA and AML. Positive response with significant remission of the loss of lung function was reported in LAM. Topical formulation of sirolimus also achieved satisfactory subjective improvement of TSC-associated angiofibroma (Luo et al., 2022). This retrospective study, in patients with TSC, provides evidence of the long-term efficacy and safety of sirolimus for controlling the size of RAHs.

For a median follow-up of 3 years, sirolimus was associated with a mean reduction of 14.6% in the thickness and 6.8% in the diameter of RAHs. Furthermore, ≥ 20% reduction in MT from baseline was observed in 20% RAHs. Results of this study are similar to previous studies in which the mean MT reduction was 13.9%–15.5%, except of their relative short-term average follow-up periods (6–8 months) compared to 3 years in the present study (Zhang et al., 2015; Marciano et al., 2018). This report adds to our knowledge regarding the long-term efficacy of sirolimus on TSC-associated RAHs. Best-corrected visual acuities were kept stable during sirolimus treatment since fovea was spared in all RAHs.

**FIGURE 3:**
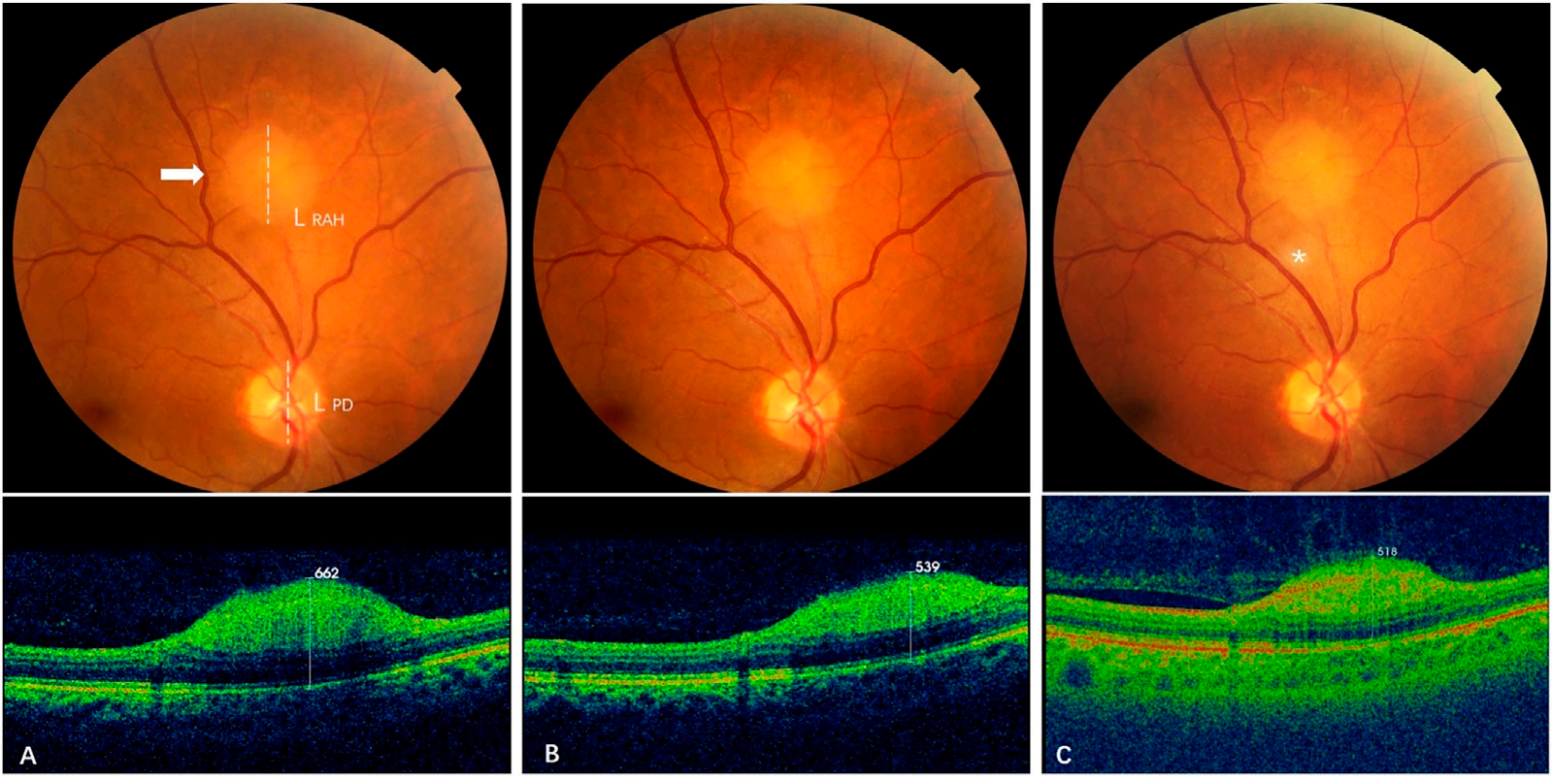
Effect of sirolimus on type 1 retinal astrocytic hamartoma over time.**(A)** At baseline, a gray-white type 1 RAH lesion (arrow) located 2 DD superior to the optic disc of the right eye was measured with a maximal thickness (MT) of 662 μm by spectral-domain optical coherence tomography (SD-OCT) and a longest base diameter (LBD) of 1.27 DD by calculating the ratio of the RAH LBD length (L _RAH,_ dashed line) to the vertical length of the disc (L_PD_, dashed line) on color fundus photography. **(B)** At 12-month follow-up (short-term), MT of the RAH decreased to 539 μm on SD-OCT, and LBD was 1.21 DD, with a slight reduction from baseline LBD. **(C)** At 28-month follow-up (long-term), the last visit, MT of the RAH further decreased to 518 μm on swept-source optical coherence tomography (SS-OCT), while the LBD remained stable at 1.22DD. The gray-white speckle (asterisk) in the center is an image artifact.

**FIGURE 4:**
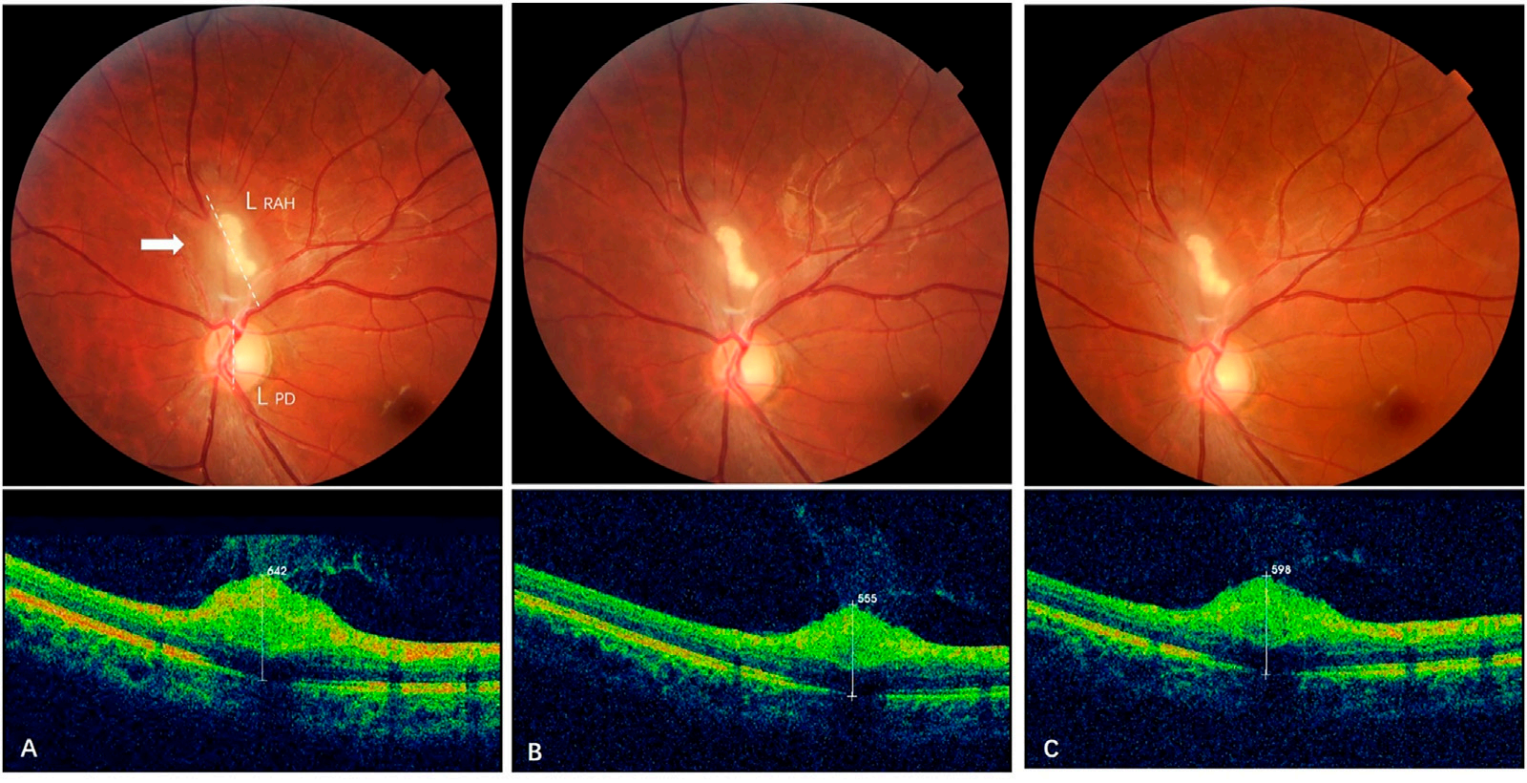
Effect of sirolimus on type 3 retinal astrocytic hamartoma over time. **(A)** At baseline, a partially calcified type 3 RAH lesion (arrow) was shown next to the superior edge of the optic disc with maximal thickness (MT) 642 μm and a longest base diameter (LBD) 1.51 DD (L _RAH_, dashed line/L_PD_, dashed line). **(B)** At 6-month follow-up (short-term), MT of the RAH decreased to 565 μm on spectral-domain optical coherence tomography, and LBD generally remained the same as baseline, 1.50 DD. **(C)** At 33-month follow-up (long-term), reduction in RAH MT persisted with a mild reverse that the last MT was 598 μm, while the LBD decreased to 1.45 DD for the last visit.

Subgroup analysis was carried out to compare the long-term effects of sirolimus on RAHs with its short-term effects. It showed that the positive impacts of sirolimus on the RAH size were mainly demonstrated in the first 6–12 month treatment, with a mean reduction of 14.8% in MT and 4.7% in LBD in the short-term follow-up, and these benefits were maintained over time without achieving sustained reduction of the lesions (representative cases are shown in Figures 3, 4). Similar observations were reported with everolimus by Marciano et al. (2018) that long-term everolimus treatment did not lead to any reduction of RAH lesions, and the peak effect of everolimus on RAH lesions was possibly reached during the first months. For other manifestations of TSC, positive effects of sirolimus on renal angiomyolipoma and seizure control were reported to decline after the second year of treatment (Canpolat et al., 2018). However, sustained reduction and increasing responses (≥50% reduction of in the tumor volume) for SEGA and renal angiomyolipoma were observed over 4 years of everolimus therapy in EXIST-1, a large randomized, placebo-controlled, phase 3 trial, although majority of the responses in this study were still within the first months of treatment (Franz et al., 2016). Therefore, we can speculate sirolimus treatment could result in rapid reduction in the RAH size, especially in RAHs’ thickness, in first 6–12 months, and continuing to use sirolimus may keep ongoing benefits in the long run, possibly preventing RAH regrowth.

Sirolimus was safe and well-tolerated in long-term with only mild side effects, and the adverse effect profile was similar to our previous short-term study (Zhang et al., 2015). Oral ulcers and acnes were the most observed. Patients experiencing adverse effects associated with sirolimus were managed with symptomatic treatment or dose reduction; only one patient had a short interruption of sirolimus due to diarrhea. No adverse effects have led to treatment termination.

The limitations in this study include its retrospective design and small sample size. Fair proportion of RAH lesions was excluded for MT or LBD analysis because of the lack of eligible SD-OCT scans or fundus images, which was common when data were retrospectively collected instead of prospectively acquisition. To address the implications of exclusion, we compared the baseline information between the eligible RAHs and those ineligible, and no significant difference was found, suggesting the results may not be substantially biased. Additionally, although there was no significant correlation between RAH size reduction and RAH type, the absence of type 2 RAH and limited number of type 3 RAH, which was due to the small sample size and relatively low prevalence of calcified RAHs in Chinese, may still make an impact on our results (Zhang et al., 2020). In previous study with everolimus, it was speculated type 1 RAHs could respond better than type 2 lesions since some type 2 RAHs were observed an increase in the dimension after 6-month everolimus treatment (Marciano et al., 2018). The effects of mTOR inhibitors on different types of RAHs need to be investigated in a larger study.

In summary, sirolimus therapy is effective at reducing the size of TSC-associated RAHs for over 2 years, with only mild adverse events during the follow-up. RAH response to sirolimus could be divided into two stages, the rapid reduction phase within first 6–12 months and the following maintenance phase in which persistent use of sirolimus possibly prevents tumor regrowth. Continuous monitoring of RAH progression and adverse events is required for ongoing treatment with sirolimus in TSC patients.

## Data Availability

The original contributions presented in the study are included in the article/Supplementary Material; further inquiries can be directed to the corresponding author.

## Abbreviations

DD, disc diameter; LBD, longest base diameter; MT, maximal thickness.
