# Supplementary material for: Long-term efficacy and safety of sirolimus for retinal astrocytic hamartoma associated with tuberous sclerosis complex
Source: Front Cell Dev Biol. 2022 Nov 18;10:973845. doi: 10.3389/fcell.2022.973845 (PMC9716018; doi:10.3389/fcell.2022.973845)
Supplement: Supplementary file 1 [file Table1.DOCX]

**Supplementary Table 1. Thirty retinal astrocytic hamartoma lesions eligible for analysis of maximal thickness on optical coherence tomography**

| **Patient No./sex/age,y^a^** | **Follow-up period, mo** | **RAH No.** | **RAH**  **type** | **RAH**  **location^b^** | **MT^c^** | | |
| --- | --- | --- | --- | --- | --- | --- | --- |
|  |  |  |  |  | **MT 1, μm** | **MT2, μm** | - **MT，%** |
| 1/M/24 | 31 | 1 | type 1 | superotemporal | 627 | 511 | -18.5% |
|  |  | 2 | type 1 | superotemporal | 395 | 331 | -16.2% |
| 2/M/13 | 47 | 1 | type 1 | perifoveal | 479 | 439 | -8.4% |
|  |  | 2 | type 1 | superotemporal | 571 | 471 | -17.5% |
|  |  | 3 | type 1 | inferonasal | 878 | 741 | -15.6% |
| 3/M/16 | 33 | 1 | type 1 | superotemporal | 471 | 435 | -7.6% |
|  |  | 2 | type 3 | superonasal | 667 | 598 | -10.3% |
| 4/F/33 | 55 | 1 | type 1 | superotemporal | 467 | 431 | -7.7% |
|  |  | 2 | type 1 | superotemporal | 407 | 379 | -6.9% |
| 5/F/19 | 31 | 1 | type 1 | superonasal | 591 | 475 | -19.6% |
|  |  | 2 | type 1 | superotemporal | 632 | 495 | -21.7% |
| 6/F/25 | 32 | 1 | type 1 | perifoveal | 369 | 339 | -8.1% |
| 7/M/13 | 36 | 1 | type 1 | superotemporal | 555 | 498 | -10.3% |
|  |  | 2 | type 1 | inferotemporal | 697 | 549 | -21.2% |
|  |  | 3 | type 1 | superotemporal | 491 | 410 | -16.5% |
|  |  | 4 | type 1 | inferotemporal | 686 | 558 | -18.7% |
|  |  | 5 | type 1 | inferonasal | 599 | 537 | -10.4% |
| 8/M/25 | 49 | 1 | type 3 | superotemporal | 570 | 460 | -19.3% |
|  |  | 2 | type 3 | superotemporal | 515 | 442 | -14.2% |
|  |  | 3 | type 1 | superotemporal | 375 | 291 | -22.4% |
| 9/F/24 | 36 | 1 | type 1 | superonasal | 590 | 458 | -22.4% |
|  |  | 2 | type 1 | inferotemporal | 666 | 535 | -19.7% |
|  |  | 3 | type 1 | superonasal | 571 | 451 | -21.0% |
| 10/F/20 | 50 | 1 | type 1 | perifoveal | 491 | 418 | -14.9% |
| 11/M/27 | 37 | 1 | type 1 | inferotemporal | 527 | 474 | -10.1% |
|  |  | 2 | type 1 | inferonasal | 512 | 478 | -6.6% |
|  |  | 3 | type 1 | perifoveal | 391 | 347 | -11.3% |
| 12/F/42 | 28 | 1 | type 1 | superotemporal | 662 | 518 | -21.8% |
|  |  | 2 | type 1 | inferonasal | 502 | 463 | -7.8% |
| 13/F/39 | 42 | 1 | type 3 | superotemporal | 482 | 426 | -11.6% |
| Abbreviations: RAH, retinal astrocytic hamartoma; M, male; F, female; MT, maximal thickness.  ^a^ Age represents age at baseline visit. The baseline visit was performed within 1 month before or after the treatment of sirolimus.  ^b^ RAHs were divided into 6 quadrants based on the location of the lesions: perifoveal (tumor distance to fovea within 3 millimeters), peripapillary (partially on the optic disc), superotemporal, inferotemporal, superonasal and inferotemporal.  ^c^ MT1 represents MT at baseline. MT2 represents MT at the last visit after the treatment with sirolimus for at least 2 years. △ MT stands for the change of maximal thickness from baseline during the follow-up. | | | | | | | |
