# Supplementary material for: Long-term efficacy and safety of sirolimus for retinal astrocytic hamartoma associated with tuberous sclerosis complex
Source: Front Cell Dev Biol. 2022 Nov 18;10:973845. doi: 10.3389/fcell.2022.973845 (PMC9716018; doi:10.3389/fcell.2022.973845)
Supplement: Supplementary file 2 [file Table2.DOCX]

**Supplementary Table 2. Comparison between RAHs eligible and ineligible for maximal thickness analysis**

| Characteristics | RAH eligible for MT analysis (N=30) | RAH ineligible for MT analysis (N=29) | *P* value |
| --- | --- | --- | --- |
| Patient age, mean (SD), y^a^ | 22.8(8.7) | 25.4(8.2) | 0.24^c^ |
| Follow-up period, mean (SD), mo^b^ | 38.9(8.0) | 37.0(5.3) | 0.30^c^ |
| RAH type |  |  | 0.35^d^ |
| type 1 | 26 | 28 |  |
| type 2 | 0 | 0 |  |
| type 3 | 4 | 1 |  |
| RAH location |  |  | 0.48^d^ |
| perifoveal | 4 | 4 |  |
| peripapilary | 0 | 2 |  |
| superotemporal | 14 | 7 |  |
| superonasal | 4 | 5 |  |
| inferotemporal | 4 | 6 |  |
| inferonasal | 4 | 5 |  |
| Abbreviations: RAH, retinal astrocytic hamartoma; MT, maximal thickness.  ^a^ The patient age was weighted by the number of RAH lesions.  ^b^ The follow-up period was weighted by the number of RAH lesions.  ^c^ One-way ANOVA  ^d^ Fisher exact test | | | |
