# Supplementary material for: Long-term efficacy and safety of sirolimus for retinal astrocytic hamartoma associated with tuberous sclerosis complex
Source: Front Cell Dev Biol. 2022 Nov 18;10:973845. doi: 10.3389/fcell.2022.973845 (PMC9716018; doi:10.3389/fcell.2022.973845)
Supplement: Supplementary file 3 [file Table3.DOCX]

**Supplementary Table 3. Forty-two retinal astrocytic hamartoma lesions eligible for analysis of longest base diameter on color fundus photography**

| **Patient No./sex/age,y^a^** | **Follow-up period, mo** | **RAH No.** | **RAH**  **type** | **RAH**  **location^b^** | **LBD^c^** | | |
| --- | --- | --- | --- | --- | --- | --- | --- |
|  |  |  |  |  | **LBD 1, DD** | **LBD 2, DD** | - **LBD，%** |
| 1/M/24 | 31 | 1 | type 1 | superotemporal | 0.46 | 0.30 | -34.5% |
|  |  | 2 | type 1 | superotemporal | 0.21 | 0.13 | -39.3% |
| 2/M/13 | 47 | 1 | type 1 | perifoveal | 0.45 | 0.51 | +13.8% |
|  |  | 2 | type 1 | superotemporal | 1.01 | 0.99 | -1.4% |
|  |  | 3 | type 1 | inferonasal | 0.72 | 0.74 | +3.9% |
| 3/M/16 | 33 | 1 | type 3 | superonasal | 1.51 | 1.46 | -3.4% |
| 4/F/33 | 55 | 1 | type 1 | superotemporal | 0.60 | 0.54 | -9.9% |
|  |  | 2 | type 1 | superotemporal | 0.51 | 0.41 | -18.5% |
| 5/F/19 | 31 | 1 | type 1 | superonasal | 0.72 | 0.73 | +0.4% |
|  |  | 2 | type 1 | superotemporal | 1.24 | 1.24 | -0.5% |
| 6/F/25 | 32 | 1 | type 1 | perifoveal | 0.21 | 0.22 | +5.4% |
| 7/M/13 | 36 | 1 | type 1 | peripapillary | 1.26 | 1.32 | +4.7% |
|  |  | 2 | type 1 | superotemporal | 1.52 | 1.29 | -15.5% |
|  |  | 3 | type 1 | inferotemporal | 0.88 | 0.84 | -4.8% |
|  |  | 4 | type 1 | superotemporal | 1.75 | 1.74 | -0.5% |
|  |  | 5 | type 1 | inferonasal | 0.64 | 0.67 | +5.2% |
|  |  | 6 | type 1 | superotemporal | 0.74 | 0.58 | -20.8% |
|  |  | 7 | type 1 | inferotemporal | 0.54 | 0.55 | +0.7% |
|  |  | 8 | type 1 | inferonasal | 1.43 | 1.32 | -8.1% |
| 8/M/25 | 49 | 1 | type 3 | superotemporal | 1.21 | 1.13 | -6.5% |
|  |  | 2 | type 3 | superotemporal | 0.69 | 0.59 | -14.9% |
|  |  | 3 | type 1 | superotemporal | 0.28 | 0.28 | +2.5% |
| 9/F/24 | 36 | 1 | type 1 | superonasal | 1.03 | 0.94 | -8.8% |
|  |  | 2 | type 1 | inferotemporal | 0.89 | 0.81 | -8.9% |
|  |  | 3 | type 1 | superonasal | 0.83 | 0.72 | -12.2% |
| 10/F/20 | 50 | 1 | type 1 | perifoveal | 0.98 | 0.89 | -9.5% |
|  |  | 2 | type 1 | superonasal | 1.00 | 0.89 | -10.3% |
| 11/M/27 | 37 | 1 | type 1 | inferotemporal | 0.68 | 0.65 | -5.0% |
|  |  | 2 | type 1 | inferonasal | 1.02 | 0.93 | -9.4% |
|  |  | 3 | type 1 | superonasal | 0.40 | 0.33 | -17.2% |
|  |  | 4 | type 1 | perifoveal | 1.34 | 1.22 | -9.1% |
|  |  | 5 | type 1 | peripapillary | 0.74 | 0.69 | -6.8% |
|  |  | 6 | type 1 | perifoveal | 0.48 | 0.44 | -8.4% |
|  |  | 7 | type 1 | superotemporal | 0.72 | 0.60 | -16.1% |
|  |  | 8 | type 1 | superotemporal | 0.88 | 0.76 | -13.2% |
|  |  | 9 | type 1 | superotemporal | 0.79 | 0.68 | -13.7% |
|  |  | 10 | type 1 | inferonasal | 0.68 | 0.65 | -4.5% |
| 12/F/42 | 28 | 1 | type 1 | superotemporal | 1.27 | 1.23 | -3.2% |

**Supplemental Table 3. Forty-two retinal astrocytic hamartoma lesions eligible for analysis of longest base diameter on color fundus photography (continued)**

| **Patient No./sex/age,y^a^** | **Follow-up period, mo** | **RAH No.** | **RAH**  **type** | **RAH**  **location^b^** | **LBD^c^** | | |
| --- | --- | --- | --- | --- | --- | --- | --- |
|  |  |  |  |  | **LBD 1, DD** | **LBD 2, DD** | - **LBD，%** |
|  |  | 2 | type 1 | superonasal | 0.51 | 0.52 | +1.7% |
|  |  | 3 | type 1 | superotemporal | 0.13 | 0.13 | -1.0% |
|  |  | 4 | type 1 | inferotemporal | 0.51 | 0.57 | +10.5% |
| 13/F/39 | 42 | 1 | type 3 | superotemporal | 0.81 | 0.82 | +1.3% |
| Abbreviations: RAH, retinal astrocytic hamartoma; M, male; F, female; LBD, longest base diameter; DD, disc diameter.  ^a^ Age represents age at baseline visit. The baseline visit was performed within 1 month before or after the treatment of sirolimus.  ^b^ RAHs were divided into 6 quadrants based on the location of the lesions: perifoveal (tumor distance to fovea within 3 millimeters), peripapillary (partially on the optic disc), superotemporal, inferotemporal, superonasal and inferotemporal.  ^c^ LBD 1 represents LBD at baseline. LBD 2 represents LBD at the last visit after the treatment with sirolimus for at least 2 years. △ LBD stands for the change of longest base diameter from baseline during the follow-up. | | | | | | | |
