# Supplementary material for: Long-term efficacy and safety of sirolimus for retinal astrocytic hamartoma associated with tuberous sclerosis complex
Source: Front Cell Dev Biol. 2022 Nov 18;10:973845. doi: 10.3389/fcell.2022.973845 (PMC9716018; doi:10.3389/fcell.2022.973845)
Supplement: Supplementary file 4 [file Table4.DOCX]

**Supplementary Table 4. Comparison between RAHs eligible and ineligible for longest base diameter analysis**

| Characteristics | RAH eligible for LBD analysis (N=42) | RAH ineligible for LBD analysis (N=17) | *P* value |
| --- | --- | --- | --- |
| Patient age, mean (SD), y^a^ | 23.8(8.9) | 24.8(7.5) | 0.70^c^ |
| Follow-up period, mean (SD), mo^b^ | 38.3(7.3) | 37.3(5.4) | 0.62^c^ |
| RAH type |  |  | 0.55^d^ |
| type 1 | 38 | 16 |  |
| type 2 | 0 | 0 |  |
| type 3 | 4 | 1 |  |
| RAH location |  |  | 0.24^d^ |
| perifoveal | 5 | 3 |  |
| peripapilary | 2 | 0 |  |
| superotemporal | 18 | 3 |  |
| superonasal | 7 | 2 |  |
| inferotemporal | 5 | 5 |  |
| inferonasal | 5 | 4 |  |
| Abbreviations: RAH, retinal astrocytic hamartoma; LBD, longest base diameter.  ^a^ The patient age was weighted by the number of RAH lesions.  ^b^ The follow-up period was weighted by the number of RAH lesions.  ^c^ One-way ANOVA  ^d^ Fisher exact test | | | |
